# Momentum contrast-enhanced multimodal representation learning for drug synergy prediction

**DOI:** 10.1093/bioinformatics/btag530

**Published:** 2026-07-17

**Authors:** Yu Gu, Xindi Huang, Lifen Shi, Leying Chen, Guihua Duan, Cheng Yan

**Affiliations:** School of Informatics, Hunan University of Chinese Medicine, Changsha 410208, China; School of Informatics, Hunan University of Chinese Medicine, Changsha 410208, China; School of Informatics, Hunan University of Chinese Medicine, Changsha 410208, China; School of Informatics, Hunan University of Chinese Medicine, Changsha 410208, China; School of Computer Science and Engineering, Central South University, Changsha 410083, China; School of Informatics, Hunan University of Chinese Medicine, Changsha 410208, China

## Abstract

**Motivation:**

Accurate prediction of synergistic drug combinations can accelerate anticancer combination discovery. Existing methods inadequately model higher order drug–drug–cell-line interactions and drug–disease associations and remain sensitive to sparse and noisy multiomics data, limiting generalization to unseen cell lines and drug combinations.

**Results:**

We present Momentum Contrast (MoCo)-MultiSynergy, a multimodal framework that combines modality-specific momentum contrastive learning with heterogeneous hypergraph modeling. The hypergraph represents synergistic drug–drug–cell-line triplets and drug–disease associations, while gated residual propagation refines node representations. MoCo modules regularize encoded drug and cell-line representations using latent feature masking and Gaussian perturbation. On the O’Neil and NCI-ALMANAC datasets, MoCo-MultiSynergy achieves the highest AUROC and AUPRC across the evaluated settings, with the largest gains when generalizing to unseen cell lines and drug combinations.

**Availability and implementation:**

Source code is available at https://github.com/27167199/MoCo-MultiSynergy.

## 1 Introduction

Identifying synergistic drug combinations is important for precision oncology because rational combinations can improve efficacy, delay resistance, and reduce dose-limiting toxicity. Large-scale drug-combination screening datasets, such as NCI-ALMANAC and O’Neil (O’Neil *et al.* 2016, Holbeck *et al.* 2017), provide valuable resources for studying drug synergy. However, these datasets cover only a small fraction of the vast combinatorial space of possible drug pairs, making exhaustive experimental screening impractical. Computational approaches have evolved from feature-based deep neural networks (Preuer *et al.* 2018) to graph- and hypergraph-based models that explicitly represent molecular structures and cellular contexts (Liu *et al.* 2022, Wu et al. 2024). Recent methods have also explored multitask learning, multiview representation learning, multisource data integration and few-shot learning for drug synergy prediction. MARSY (El Khili *et al.* 2023) jointly predicts drug-combination synergy and single-drug responses across multiple cell lines. JointSyn (Li *et al.* 2024) integrates drug structural and cell-line transcriptomic views for personalized synergy prediction. MultiSyn (Jin *et al.* 2025) combines protein–protein interaction networks, multiomics profiles and pharmacophore information, while few-shot approaches have been developed for data-limited cellular contexts (Feng *et al.* 2025). These advances demonstrate the value of integrating heterogeneous biomedical information; however, the robustness of multimodal representations to noisy and incomplete inputs remains underexplored in higher order drug synergy models.

Despite these advances, two challenges remain. First, drug synergy is a context-dependent higher order phenomenon involving drug pairs, cell lines, and disease backgrounds. Models based mainly on pairwise drug representations or direct feature concatenation may not fully capture these relationships, limiting their ability to generalize to unseen-drug combinations and cellular contexts. Second, drug synergy datasets and multiomics profiles contain missing measurements, technical variation and biological heterogeneity. Models trained mainly with supervised synergy labels may therefore learn dataset-specific patterns rather than robust biological representations. To address these challenges, Momentum Contrast (MoCo)-MultiSynergy combines modality-specific momentum contrastive learning with heterogeneous hypergraph modeling. The contrastive modules regularize encoded drug and cell-line representations using latent feature masking and Gaussian perturbation, thereby reducing sensitivity to representation noise and measurement variability. The heterogeneous hypergraph explicitly models synergistic drug–drug–cell-line interactions and drug–disease associations, while gated residual propagation refines node representations and alleviates oversmoothing.

The key contributions are three-fold: (i) modality-specific MoCo modules that regularize molecular-graph and gene-expression encoders using latent feature masking and Gaussian perturbation; (ii) integration of MoCo-enhanced representations with heterogeneous hypergraph modeling and gated residual propagation to capture higher order drug–drug–cell-line interactions and drug–disease associations; (iii) evaluation on the O’Neil and NCI-ALMANAC datasets against eight representative baselines, demonstrating improved performance when generalizing to unseen cell lines and drug combinations under challenging evaluation settings.

## 2 Materials and methods

### 2.1 Datasets and preprocessing

We integrated data from four main sources: drug synergy screening results, drug molecular structures, cancer cell-line gene expression profiles and drug–disease associations. All data were obtained from publicly available databases.

- **Drug synergy data** were collected from two large-scale pharmacological studies: the O’Neil dataset (O’Neil *et al*. 2016), which provides Loewe synergy scores for 38 drugs across 39 cell lines (23 062 drug-pair–cell-line samples), and the NCI-ALMANAC dataset (Holbeck *et al*. 2017), which reports ComboScores for 104 FDA-approved drugs tested in pairwise combinations on the NCI-60 cell-line panel (304 549 samples).
- **Drug molecular structures** were retrieved in SMILES format from PubChem (Kim *et al*. 2019) and subsequently converted into molecular graphs for feature extraction.
- **Gene expression profiles** of cancer cell lines were obtained from the COSMIC Cell Lines Project (Forbes *et al*. 2015). We extracted expression values of 651 cancer-related genes from the COSMIC Cancer Gene Census, applied ${\;\text{log}\;}_{2}$ transformation, and performed *z*-score normalization. Where available, expression profiles from the Cancer Cell Line Encyclopedia (CCLE) (Barretina *et al*. 2012) and Genomics of Drug Sensitivity in Cancer (Garnett *et al*. 2012) were used as supplementary sources to improve cell-line coverage. Profiles from all sources were restricted to the same 651-gene panel and processed using the same ${\;\text{log}\;}_{2}$ transformation and *z*-score normalization.
- **Drug–disease associations** were extracted from PrimeKG (Chandak *et al*. 2023), a multimodal knowledge graph that integrates several biomedical resources, including DisGeNET (Piñero *et al*. 2020) and DrugBank (Wishart *et al*. 2018), and captures diverse drug–disease relationships, including indications, contraindications, and off-label uses.

Both synergy datasets underwent thorough preprocessing: cell lines without gene expression profiles and drugs lacking valid SMILES strings were removed. After filtering, the NCI-ALMANAC dataset contained 74 139 samples (87 drugs, 55 cell lines), and the O’Neil dataset contained 18 950 samples (38 drugs, 39 cell lines). Drug–disease associations were further filtered to retain only drugs present in the respective synergy sets, resulting in 82 diseases linked to 37 drugs for NCI-ALMANAC and 42 diseases linked to 12 drugs for O’Neil. Dataset statistics after preprocessing are summarized in Table 1.

**Table 1:** Statistics of the preprocessed synergy datasets.

Dataset	Drugs	Cell lines	Samples
NCI-ALMANAC	87	55	74 139
O’Neil	38	39	18 950

### 2.2 Proposed framework

#### 2.2.1 Overview

**Figure 1:**
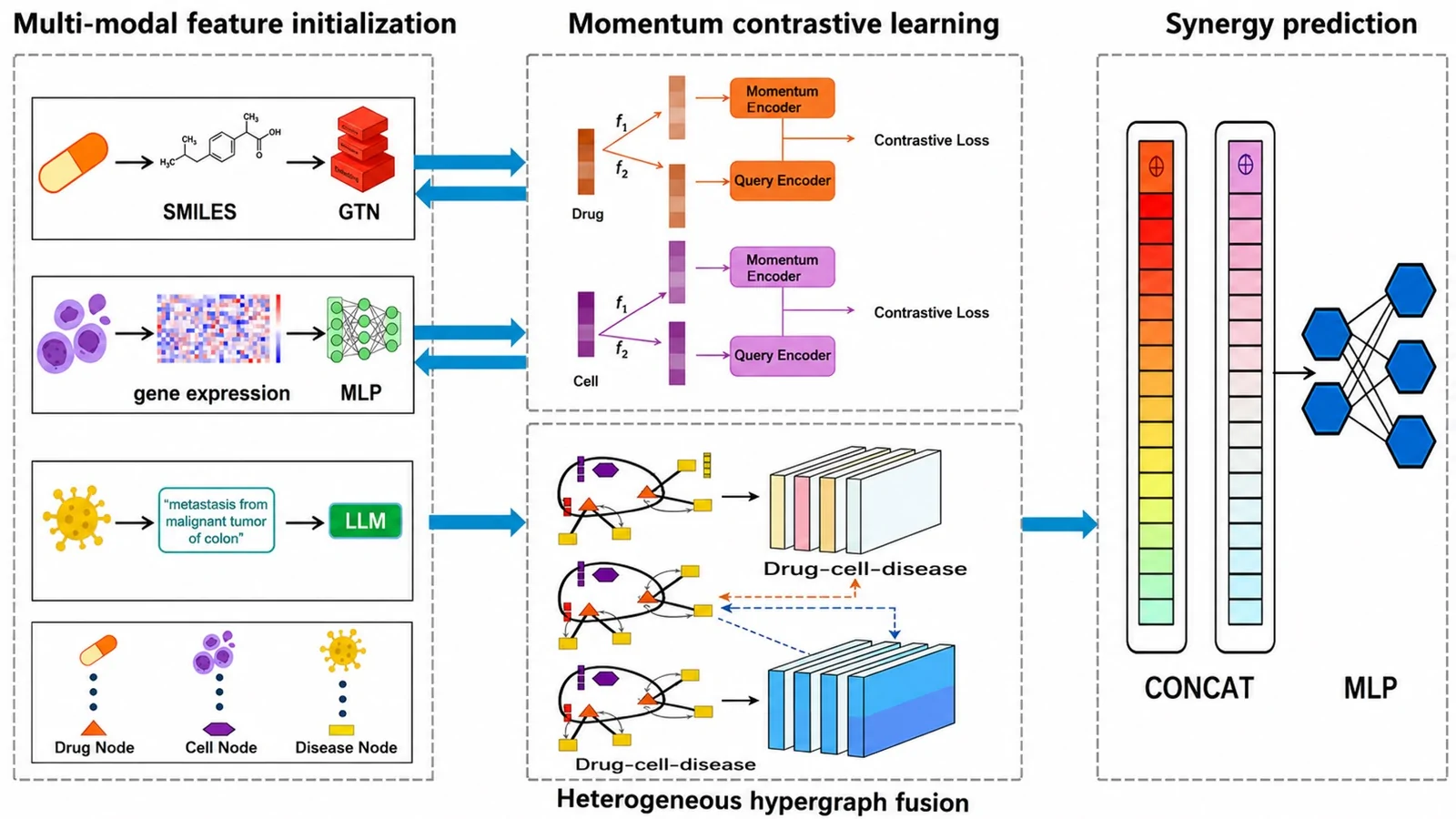
Overview of MoCo-MultiSynergy. The four stages are: (i) multimodal feature initialization; (ii) modality-specific momentum contrastive learning; (iii) heterogeneous hypergraph fusion of drug–drug–cell-line and drug–disease relations; and (iv) synergy prediction from the refined representations of two drugs and a cell line.

MoCo-MultiSynergy comprises four stages (Fig. 1). First, drug molecular graphs, cell-line gene expression profiles, and available disease features are mapped into a shared 256-dimensional latent space. Second, modality-specific MoCo modules regularize the encoded drug and cell-line representations by enforcing consistency between augmented latent views. Third, the resulting embeddings are propagated through a heterogeneous hypergraph containing synergistic drug–drug–cell-line hyperedges and drug–disease associations and refined through gated residual updates. Finally, the refined representations of two drugs and the corresponding cell line are concatenated to predict synergy.

#### 2.2.2 Multimodal feature initialization

MoCo-MultiSynergy uses modality-specific encoders because drug molecules, cell-line expression profiles, and disease concepts differ substantially in structure and dimensionality. Each encoder maps its input into a shared 256-dimensional latent space before heterogeneous hypergraph fusion.

For the drug modality, each SMILES string is converted into a molecular graph $G_{i}=(V_{i},E_{i})$, where atoms are represented as nodes and chemical bonds as edges. Let $X_{i}^{d}$ denote the initial atom-feature matrix. A three-layer TransformerConv encoder (Shi *et al.* 2020) with four attention heads aggregates atom-level information. ReLU activation and BatchNorm are applied after each layer, and residual connections are introduced in the later layers to preserve lower level molecular information. Let $\phi_{d}^{\left(\ell \right)}$ denote a TransformerConv layer followed by ReLU activation and BatchNorm. The first layer and the subsequent residual layers are defined as follows:


(1) $$H_{i}^{d,(1)}=\phi_{d}^{\left(1\right)}(X_{i}^{d},E_{i}),$$

(2) $$H_{i}^{d,(\ell +1)}=H_{i}^{d,(\ell )}+\phi_{d}^{\left(\ell +1\right)}(H_{i}^{d,(\ell )},E_{i}),\;\ell \in \{1,2\}.$$

Global mean pooling produces the graph-level representation $d_{i}=\mathrm{MeanPool}(H_{i}^{d,(3)})$, where global mean pooling aggregates the atom representations into a fixed-length vector. This encoder captures both local atomic interactions and higher order molecular substructures.

For the cell-line modality, each cell line is represented by a 651-dimensional gene expression vector $x_{i}^{c}$. A linear transformation with tanh activation projects the expression profile into the common latent space, after which two residual blocks refine the representation:


(3) $$c_{i}^{\left(0\right)}=\text{tanh}\left(W_{c}^{\left(0\right)}x_{i}^{c}+b_{c}^{\left(0\right)}\right),$$

(4) $$c_{i}^{\left(\ell +1\right)}=c_{i}^{\left(\ell \right)}+\phi_{c}^{\left(\ell \right)}(c_{i}^{\left(\ell \right)}),\;\ell \in \{0,1\},$$

where $\phi_{c}^{\left(\ell \right)}$ denotes a linear transformation followed by ReLU activation and dropout. The final cell-line representation is $c_{i}=c_{i}^{\left(2\right)}$. This residual design preserves transcriptomic information while learning nonlinear gene-expression patterns.

For the disease modality, each disease concept is initialized using a 768-dimensional CODER biomedical embedding $x_{i}^{s}$ (Yuan *et al.* 2022). A modality-specific projection and residual refinement map the embedding into the shared latent space:


(5) $$s_{i}=f_{s}(x_{i}^{s}),$$

where $f_{s}$ denotes the disease encoder. Disease features provide therapeutic context for subsequent hypergraph reasoning and are not subjected to contrastive augmentation.

#### 2.2.3 Momentum contrastive learning

This stage uses MoCo as an auxiliary self-supervised objective to learn discriminative drug and cell-line representations that are robust to feature perturbations. For each encoded drug or cell line, two stochastic views are generated using feature masking and Gaussian perturbation. For drugs, the perturbations are applied to encoded atom-level features before graph pooling; for cell lines, they are applied to the latent transcriptomic representation.

One augmented view is processed by a query projection head, whereas the other is processed by a momentum-updated key projection head. The key parameters are updated by exponential moving average:


(6) $$\theta_{k}^{r}=m\theta_{k}^{r}+(1-m)\theta_{q}^{r},\;r\in \{d,c\},$$

where $\theta_{q}^{r}$ and $\theta_{k}^{r}$ denote the query and key parameters for modality *r*, respectively, and the momentum coefficient is set to m=0.999. A first-in-first-out queue stores key representations from previous iterations. Unlike batch-based contrastive methods such as SimCLR (Chen *et al.* 2020) and GraphCL (You *et al.* 2020), which typically construct negative pairs from other samples in the current mini-batch, MoCo uses this dynamic queue to provide a large and consistent negative set without requiring a large mini-batch.

For modality *r*, the contrastive objective is defined using the InfoNCE loss (van den Oord *et al.* 2018):


(7) $$L_{{\text{MoCo}}_{r}}=-\text{log}\;\frac{\;\text{exp}(q_{i}^{r}\cdot k_{i}^{r+}/\tau )}{\;\text{exp}(q_{i}^{r}\cdot k_{i}^{r+}/\tau )+\sum_{n=1}^{K}\;\text{exp}\;(q_{i}^{r}\cdot k_{n}^{r-}/\tau )},$$

where $q_{i}^{r}$ and $k_{i}^{r+}$ form a positive pair from two views of the same entity, ${\left\{k_{n}^{r-}\right\}}_{n=1}^{K}$ are negative keys stored in the queue, *K* is the queue size, and the temperature is set to τ=0.07. This objective promotes consistency between views of the same entity while separating different entities, thereby regularizing both encoders.

#### 2.2.4 Heterogeneous hypergraph fusion

The initialized drug, cell-line and disease representations are integrated into a heterogeneous hypergraph. Synergistic drug–drug–cell-line triplets in the training data are represented as three-node hyperedges, enabling the model to capture the dependence of a drug combination on its cellular context. Curated drug–disease associations from PrimeKG are incorporated as auxiliary relations. Only available associations are included, and no missing drug–disease links are imputed.

The initial node-representation matrix is obtained by concatenating the three modality-specific matrices:


(8) $$X^{\left(0\right)}=\mathrm{concat}\left(X_{\text{drug}},X_{\text{cell}},X_{\text{dis}}\right).$$

Let *H* denote the hypergraph incidence matrix, *W* the diagonal hyperedge-weight matrix, and *D* and *B* the diagonal node-degree and hyperedge-degree matrices, respectively. At refinement layer ℓ, higher order information is propagated through the normalized node–hyperedge–node operator:


(9) $$Z^{\left(\ell \right)}=\mathrm{ReLU}\left(D^{-1}HWB^{-1}H^{⊤}\mathrm{BN}(X^{\left(\ell \right)})\Theta^{\left(\ell \right)}\right),$$

where $\Theta^{\left(\ell \right)}$ is the learnable transformation matrix of the ℓth hypergraph layer.

Increasing the number of hypergraph layers can cause node representations to become overly similar. To alleviate this oversmoothing effect, the node-specific gate and gated residual update are defined as follows:


(10) $$g^{\left(\ell \right)}=\sigma \left(W_{g}^{\left(\ell \right)}X^{\left(\ell \right)}+b_{g}^{\left(\ell \right)}\right),$$

(11) $$X^{\left(\ell +1\right)}=X^{\left(\ell \right)}+g^{\left(\ell \right)}⊙Z^{\left(\ell \right)}.$$

The residual connection preserves the current node representation, whereas the learned gate adaptively controls the contribution of higher order information propagated through the heterogeneous hypergraph.

#### 2.2.5 Synergy prediction

Let $X^{\left(L\right)}$ denote the final node matrix. For triplet (i,j,k), ${\tilde{d}}_{i}$, ${\tilde{d}}_{j}$, and ${\tilde{c}}_{k}$ are the rows corresponding to drugs *i* and *j* and cell line *k*, respectively. Their concatenation is passed through a multilayer perceptron to predict synergy:


(12) $${\hat{y}}_{\mathrm{ijk}}=\sigma \left(f_{\text{pred}}\left[{\tilde{d}}_{i}\|{\tilde{d}}_{j}\|{\tilde{c}}_{k}\right]\right),$$

where *i* and *j* index the drugs, *k* indexes the cell line, ‖ denotes concatenation, and $f_{\text{pred}}$ is the prediction network.

The model is jointly optimized using the supervised prediction loss, an auxiliary reconstruction loss and the two modality-specific contrastive losses:


(13) $$L_{\text{total}}=L_{\text{BCE}}+\alpha L_{\text{rec}}+\beta \left(L_{{\text{MoCo}}_{d}}+L_{{\text{MoCo}}_{c}}\right),$$

where $L_{\text{BCE}}$ is the binary cross-entropy loss for synergy prediction, $L_{\text{rec}}$ is the auxiliary reconstruction loss, and α  β control their relative contributions. The reconstruction objective uses modality-specific bilinear decoders to preserve predefined relationships among drugs, cell lines and diseases. Specifically, the drug-target matrix is derived from molecular fingerprint similarities, whereas the cell-line and disease targets are constructed from relationships among their feature profiles.

Joint optimization therefore combines predictive supervision, modality-specific structure preservation and contrastive regularization. Detailed network dimensions, augmentation procedures, queue configuration and training details are provided in Table S1, available as supplementary data at *Bioinformatics* online and Fig. S1, available as supplementary data at *Bioinformatics* online.

### 2.3 Baseline methods and evaluation metrics

To evaluate the performance of MoCo-MultiSynergy, we compared it with eight representative baseline methods:

- **DeepSynergy** (Preuer *et al*. 2018): a three-layer feedforward neural network that integrates drug chemical descriptors with cell-line gene expression profiles.
- **DTF** (Sun *et al*. 2020): a deep tensor factorization method that formulates drug synergy prediction within a tensor-based representation framework.
- **HypergraphSynergy** (Liu *et al*. 2022): a hypergraph neural network that represents drug–drug–cell-line relationships as hyperedges.
- **NHP** (Yadati *et al*. 2020): a neural hypergraph link prediction method for learning representations and predicting links in hypergraphs.
- **HERMES** (Wu *et al*. 2024): a heterogeneous hypergraph framework that integrates drug, cell-line and disease nodes with gated residual propagation.
- **MARSY** (El Khili *et al*. 2023): a multitask deep learning framework that jointly predicts drug-combination synergy and single-drug response across multiple cell lines.
- **JointSyn** (Li *et al*. 2024): a dual-view joint learning method that combines drug structural information with cell-line transcriptomic profiles for personalized drug synergy prediction.
- **MultiSyn** (Jin *et al*. 2025): a multisource information fusion framework that integrates protein interaction networks, multiomics profiles, and pharmacophore information for drug synergy prediction.

For all baselines, we used the authors’ original implementations and recommended hyperparameters where available. MARSY, JointSyn, and MultiSyn were evaluated using their publicly available implementations under the same partitioning modes and evaluation protocol as MoCo-MultiSynergy. AUROC and AUPRC were used as the primary evaluation metrics. F1 score was additionally reported for the Random-mode comparison with a random classifier, and accuracy was reported for cross-dataset validation.

### 2.4 Experimental setup

The task was formulated as binary classification. Models were trained and validated independently using the NCI-ALMANAC and O’Neil datasets. For each dataset, five-fold cross-validation was performed under three partitioning modes:

- **Random:** Samples were randomly divided into five folds.
- **CLine:** Samples were stratified by target cell line, ensuring that each validation set contained cell lines not present in the corresponding training set.
- **DrugComb:** Samples were stratified by drug combination, ensuring that each validation set contained drug combinations not observed during training, although individual drugs could overlap.

For each mode, four folds were used for training and one for validation. Results are reported as mean ± standard deviation across five folds. Statistical significance was assessed using two-sample *t*-tests based on the foldwise AUROC and AUPRC values (*P*<.05).

Beyond these three primary partitioning strategies, DrugSingle and DrugDouble were used to further evaluate generalization to previously unseen drugs. In DrugSingle, one drug in each validation combination was absent from the training set; in DrugDouble, neither drug appeared during training. In addition, bidirectional cross-dataset validation was performed using the drugs and cell lines shared by O’Neil and NCI-ALMANAC.

### 2.5 Hyperparameter configuration and sensitivity analyses

For the primary binary classification experiments, following previous studies (O’Neil *et al.* 2016, Preuer *et al.* 2018), ComboScores for NCI-ALMANAC and Loewe scores for O’Neil were binarized at a threshold of 30, with scores greater than or equal to 30 labeled as synergistic and lower scores labeled as antagonistic. Sensitivity to this label definition was evaluated on O’Neil using thresholds of {0,10,20,30,40,50}. Results for MoCo-MultiSynergy are reported in Table S2, available as supplementary data at *Bioinformatics* online, while methodwise AUROC and AUPRC comparisons are provided in Tables S3 and S4, available as supplementary data at *Bioinformatics* online and Fig. S2, available as supplementary data at *Bioinformatics* online.

**Table 2:** Performance comparison on the O’Neil dataset under the Random, Cline, and DrugComb modes.

Method	Random		CLine		DrugComb	
	AUROC	AUPRC	AUROC	AUPRC	AUROC	AUPRC
DeepSynergy	0.9060 ± 0.0062	0.4920 ± 0.0125	0.8350 ± 0.0071	0.3700 ± 0.0132	0.8100 ± 0.0078	0.4200 ± 0.0128
NHP	0.8707 ± 0.0058	0.5540 ± 0.0116	0.8200 ± 0.0069	0.3600 ± 0.0129	0.8290 ± 0.0067	0.4400 ± 0.0121
DTF	0.9138 ± 0.0052	0.5920 ± 0.0108	0.8400 ± 0.0065	0.3800 ± 0.0120	0.8200 ± 0.0068	0.4300 ± 0.0119
HypergraphSynergy	0.9230 ± 0.0045	0.6280 ± 0.0096	0.8600 ± 0.0058	0.4200 ± 0.0110	0.8620 ± 0.0055	0.4700 ± 0.0107
HERMES	0.9330 ± 0.0039	0.6647 ± 0.0088	0.8753 ± 0.0049	0.4350 ± 0.0102	0.8830 ± 0.0047	0.4900 ± 0.0100
MARSY	0.9221 ± 0.0048	0.5786 ± 0.0102	0.8806 ± 0.0055	0.5031 ± 0.0115	0.8954 ± 0.0050	0.5118 ± 0.0110
JointSyn	0.9264 ± 0.0035	0.6479 ± 0.0085	0.9158 ± 0.0038	0.6210 ± 0.0090	0.9145 ± 0.0036	0.6099 ± 0.0088
MultiSyn	0.9281 ± 0.0030	0.6674 ± 0.0075	0.9172 ± 0.0032	0.6354 ± 0.0080	0.9103 ± 0.0033	0.6276 ± 0.0082
MoCo-MultiSynergy	**0.9433** ± **0.0015**	**0.7124** ± **0.0038**	**0.9312** ± **0.0022**	**0.6708** ± **0.0045**	**0.9311** ± **0.0024**	**0.6813** ± **0.0049**

Results are reported as mean ± standard deviation over five folds. The best result in each column is shown in bold.

**Table 3:** Performance comparison on the NCI-ALMANAC dataset under the Random, Cline, and DrugComb modes.

Method	Random		CLine		DrugComb	
	AUROC	AUPRC	AUROC	AUPRC	AUROC	AUPRC
DeepSynergy	0.8350 ± 0.0058	0.4912 ± 0.0115	0.7000 ± 0.0072	0.3000 ± 0.0135	0.7600 ± 0.0068	0.3500 ± 0.0128
NHP	0.7769 ± 0.0062	0.3768 ± 0.0122	0.7400 ± 0.0065	0.3600 ± 0.0127	0.7570 ± 0.0064	0.3200 ± 0.0130
DTF	0.8238 ± 0.0055	0.4679 ± 0.0110	0.7500 ± 0.0061	0.4100 ± 0.0121	0.7500 ± 0.0060	0.3300 ± 0.0125
HypergraphSynergy	0.8530 ± 0.0045	0.5598 ± 0.0102	0.7800 ± 0.0056	0.4500 ± 0.0115	0.7800 ± 0.0054	0.3800 ± 0.0112
HERMES	0.8537 ± 0.0042	0.5619 ± 0.0098	0.7940 ± 0.0050	0.4650 ± 0.0108	0.7975 ± 0.0048	0.4300 ± 0.0105
MARSY	0.8357 ± 0.0042	0.5369 ± 0.0095	0.7768 ± 0.0058	0.4367 ± 0.0120	0.7796 ± 0.0055	0.4313 ± 0.0118
JointSyn	0.8355 ± 0.0041	0.5197 ± 0.0099	0.8223 ± 0.0039	0.4968 ± 0.0098	0.8313 ± 0.0031	0.4733 ± 0.0107
MultiSyn	0.8354 ± 0.0040	0.5148 ± 0.0100	0.8241 ± 0.0038	0.5041 ± 0.0095	0.8199 ± 0.0036	0.4809 ± 0.0105
MoCo-MultiSynergy	**0.8556** ± **0.0018**	**0.5675** ± **0.0042**	**0.8451** ± **0.0023**	**0.5454** ± **0.0050**	**0.8446** ± **0.0021**	**0.5403** ± **0.0055**

Results are reported as mean ± standard deviation over five folds. The best result in each column is shown in bold.

**Table 4:** Ablation study results (AUROC) on the O’Neil dataset under the Random, Cline, and DrugComb modes.

Strategy	Random	CLine	DrugComb
**MoCo-MultiSynergy**	**0.9433** ± **0.0015**	**0.9312** ± **0.0022**	**0.9311** ± **0.0024**
w/o MoCo (drug + cell)	0.9330 ± 0.0032	0.8753 ± 0.0038	0.8830 ± 0.0047
w/o MoCo (drug)	0.9418 ± 0.0023	0.9301 ± 0.0032	0.9304 ± 0.0039
w/o MoCo (cell)	0.9426 ± 0.0019	0.9300 ± 0.0026	0.9301 ± 0.0036
w/o gated residual	0.9374 ± 0.0027	0.9219 ± 0.0040	0.9215 ± 0.0026
w/o hypergraph	0.9348 ± 0.0008	0.9197 ± 0.0017	0.9201 ± 0.0011

All values are reported as mean ± standard deviation over five folds and bold values indicate the best performance.

The main hyperparameters, including the learning rate, weight decay, number of attention heads, number of hypergraph layers, loss weights, MoCo temperature, and queue size, were selected according to mean validation AUROC. The complete search space and selected configuration are provided in Table S1, available as supplementary data at *Bioinformatics* online.

Training comprised 100 epochs of modality-encoder pretraining followed by joint optimization for up to 3000 epochs using Adam. The InfoNCE temperature was fixed at τ=0.07 throughout training. Additional architecture and training details are provided in the Supplementary Methods, available as supplementary data at *Bioinformatics* online. Temperature sensitivity was evaluated on O’Neil under the Random mode using {0.01,0.03,0.05,0.07,0.10,0.20,0.50} (Table S5, available as supplementary data at *Bioinformatics* online).

**Table 5:** Top 10 paclitaxel-containing combinations ranked by MoCo-MultiSynergy.

Drug A	Drug B	Cell line	Predicted score	Publications
Paclitaxel	MK-8669	SK-OV-3	0.994538	Sessa *et al.* (2010)
Paclitaxel	MRK-003	SK-OV-3	0.994079	Groeneweg *et al.* (2014)
Paclitaxel	AZD1775	MSTO-211H	0.989496	Oza *et al.* (2020)
Paclitaxel	MK-8669	MSTO-211H	0.988871	Sessa *et al.* (2010)
Paclitaxel	MK-2206	MSTO-211H	0.986012	Gonzalez-Angulo *et al.* (2015)
Paclitaxel	AZD1775	NCI-H520	0.985859	Oza *et al.* (2020)
Paclitaxel	MK-5108	SK-OV-3	0.985845	NA
Paclitaxel	AZD1775	A2780	0.985639	Oza *et al.* (2020)
Paclitaxel	MK-2206	NCI-H520	0.983492	Gonzalez-Angulo *et al.* (2015)
Paclitaxel	Dexamethasone	SK-OV-3	0.983453	NA

Publications refer to studies of the same drug pair or closely related treatment regimens; NA indicates that no relevant publication support was identified.

## 3 Results

### 3.1 Performance comparison

**Figure 2:**
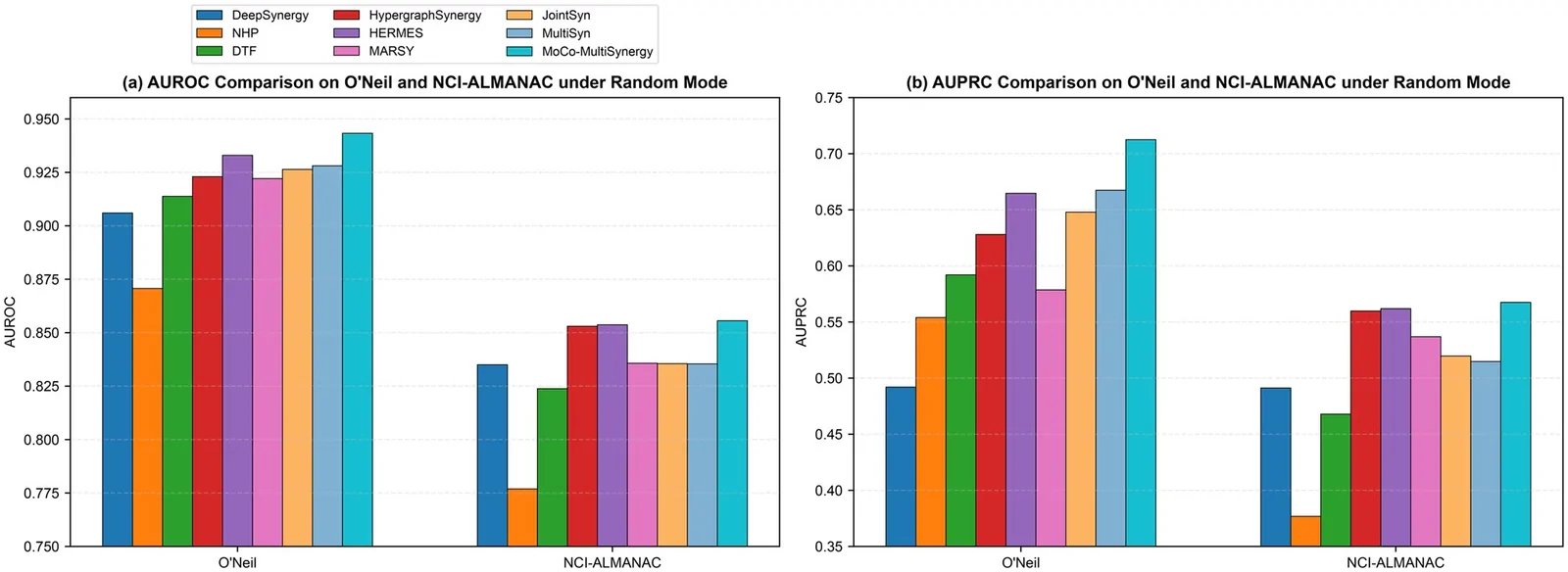
Performance comparison under the Random mode on the O’Neil and NCI-ALMANAC datasets. (a) AUROC comparison; (b) AUPRC comparison. Complete numerical results are reported in Tables 2 and 3.

**Figure 3:**
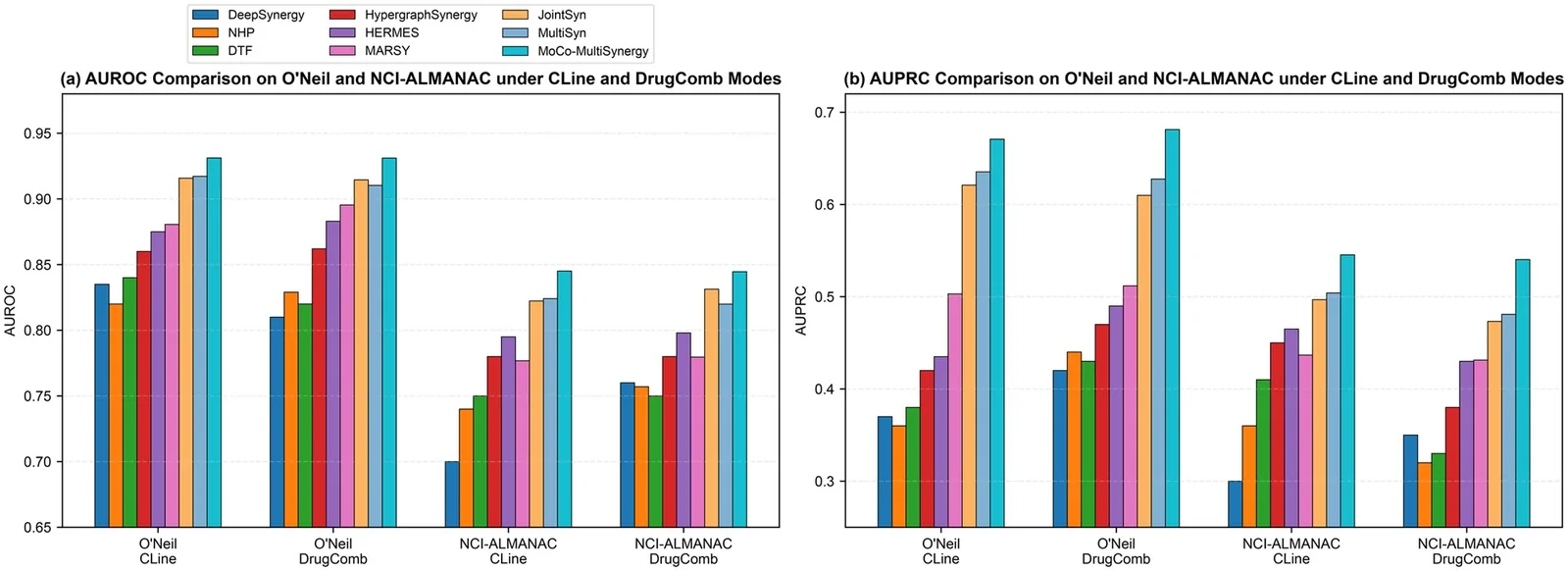
Performance comparison under the CLine and DrugComb modes. (a) AUROC comparison across O’Neil-CLine, O’Neil-DrugComb, NCI-ALMANAC-Cline, and NCI-ALMANAC-DrugComb; (b) corresponding AUPRC comparison. Complete numerical results are reported in Tables 2 and 3.

MoCo-MultiSynergy was compared with eight baselines on the O’Neil and NCI-ALMANAC datasets under the Random, Cline, and DrugComb modes (Tables 2 and 3). The Random results are visualized in Fig. 2, and the CLine and DrugComb results in Fig. 3.

Across both datasets, MoCo-MultiSynergy achieved the highest AUROC and AUPRC in all three modes (Tables 2 and 3). The improvements were modest under the Random mode, particularly on NCI-ALMANAC, where MoCo-MultiSynergy achieved an AUROC of 0.8556 and an AUPRC of 0.5675, compared with an AUROC of 0.8537 and an AUPRC of 0.5619 for HERMES.

Larger improvements were observed under the CLine and DrugComb modes. On O’Neil, MoCo-MultiSynergy achieved an AUROC of 0.9312 and an AUPRC of 0.6708 under the CLine mode, whereas the strongest baseline, MultiSyn, achieved 0.9172 and 0.6354, respectively. Under the DrugComb mode, MoCo-MultiSynergy achieved an AUROC of 0.9311 and an AUPRC of 0.6813. The highest baseline AUROC was 0.9145 for JointSyn, whereas the highest baseline AUPRC was 0.6276 for MultiSyn. On NCI-ALMANAC, MoCo-MultiSynergy achieved an AUROC of 0.8451 and an AUPRC of 0.5454 under the CLine mode, compared with 0.8241 and 0.5041 for MultiSyn. Under the DrugComb mode, MoCo-MultiSynergy achieved an AUROC of 0.8446 and an AUPRC of 0.5403. The highest baseline AUROC was 0.8313 for JointSyn, and the highest baseline AUPRC was 0.4809 for MultiSyn. The performance advantage was therefore clearest when generalizing to unseen cell lines and drug combinations.

Comparisons with the random classifier showed that all learned models outperformed the random baseline under the Random mode, with MoCo-MultiSynergy achieving the strongest overall results on both datasets (Tables S6 and S7, available as supplementary data at *Bioinformatics* online). Statistical comparisons with HERMES further showed significant improvements on O’Neil under all three modes and on NCI-ALMANAC under the CLine and DrugComb modes; the differences under the NCI-ALMANAC Random mode were not statistically significant (Tables S8 and S9, available as supplementary data at *Bioinformatics* online).

**Table 6:** Top 10 carboplatin-containing combinations ranked by MoCo-MultiSynergy.

Drug A	Drug B	Cell line	Predicted score	Publications
Carboplatin	BEZ-235	NCI-H520	0.990436	NA
Carboplatin	MK-8669	A2780	0.989488	Chon *et al.* (2017)
Carboplatin	BEZ-235	A2780	0.988055	NA
Carboplatin	AZD1775	OVCAR-3	0.979448	Leijen *et al.* (2016), Oza *et al.* (2020)
Carboplatin	Bortezomib	MSTO-211H	0.969157	Aghajanian *et al.* (2005)
Carboplatin	AZD1775	A2780	0.967725	Leijen *et al.* (2016), Oza *et al.* (2020)
Carboplatin	MK-8669	SK-OV-3	0.965579	Chon *et al.* (2017)
Carboplatin	MK-8669	A427	0.965129	Chon *et al.* (2017)
Carboplatin	AZD1775	A427	0.961204	Leijen *et al.* (2016), Oza *et al.* (2020)
Carboplatin	AZD1775	MSTO-211H	0.948358	Leijen *et al.* (2016), Oza *et al.* (2020)

Publications refer to studies of the same drug pair or closely related treatment regimens; NA indicates that no relevant publication support was identified.

### 3.2 Unseen-drug and cross-dataset evaluation

To assess generalization beyond the drugs and data distributions observed during training, we conducted two complementary experiments. First, leave-drug-out evaluation was performed on both the O’Neil and NCI-ALMANAC datasets under the DrugSingle and DrugDouble modes. Second, bidirectional cross-dataset validation was performed by training on one dataset and testing on the other using the drugs and cell lines shared by the two datasets.

In the leave-drug-out evaluation, MoCo-MultiSynergy achieved an AUROC of 0.8083 and an AUPRC of 0.4572 on O’Neil under the DrugSingle mode. Under the more stringent DrugDouble mode, the corresponding values were 0.7648 and 0.4058. On NCI-ALMANAC, the model achieved an AUROC of 0.7531 and an AUPRC of 0.3105 in the DrugSingle mode, and an AUROC of 0.7135 and an AUPRC of 0.2793 in the DrugDouble mode. MoCo-MultiSynergy outperformed HERMES and HypergraphSynergy in both modes on the two datasets. Complete leave-drug-out results are reported in Tables S10 and S11, available as supplementary data at *Bioinformatics* online.

Cross-dataset validation provided a more stringent evaluation under differences in synergy definitions and experimental distributions. When trained on O’Neil and evaluated on NCI-ALMANAC, MoCo-MultiSynergy achieved an AUROC of 0.5922 and an accuracy of 0.7829. In the reverse direction, training on NCI-ALMANAC and testing on O’Neil resulted in an AUROC of 0.6879 and an accuracy of 0.8023. Although these values were lower than those obtained in within-dataset evaluation, the model retained discriminative information across independently generated drug-combination datasets. Complete cross-dataset results are reported in Table S12, available as supplementary data at *Bioinformatics* online.

### 3.3 Ablation study

We conducted three groups of ablation experiments to examine the contributions of the principal model components and design choices. First, component ablations removed the modality-specific MoCo modules, the heterogeneous hypergraph, or the gated residual mechanism across the Random, CLine and DrugComb modes. Second, graph-level ablations removed or shuffled drug–disease edges to assess the influence of incomplete disease annotations. Third, encoder ablations replaced TransformerConv with GCN or GAT to evaluate the effect of the drug molecular encoder.

In the component ablation analysis, the complete model achieved the highest AUROC and AUPRC across all three modes (Tables S4 and S13, available as supplementary data at *Bioinformatics* online). Removing both modality-specific MoCo modules caused the largest reduction: under the CLine and DrugComb modes, AUROC decreased from 0.9312 to 0.8753 and from 0.9311 to 0.8830, while AUPRC decreased from 0.6708 to 0.4035 and from 0.6813 to 0.4900, respectively. Removing either MoCo module alone produced smaller reductions, whereas removing the heterogeneous hypergraph or gated residual mechanism also reduced performance across all three modes.

The graph-level ablations showed that removing all drug–disease edges or randomly permuting their assignments caused only moderate performance changes relative to the complete model (Table S14, available as supplementary data at *Bioinformatics* online). These results suggest that the performance gains were not driven solely by the availability or assignment of drug–disease edges.

In the encoder ablation, TransformerConv was replaced with GCN and GAT, while all remaining components were held fixed. TransformerConv achieved the highest AUROC and AUPRC under the Random, Cline, and DrugComb modes (Table S15, available as supplementary data at *Bioinformatics* online), supporting the use of attention-based molecular graph encoding in MoCo-MultiSynergy.

### 3.4 Drug-centric case study

To assess the biological relevance of the predictions, we selected paclitaxel and carboplatin as clinically relevant anchor drugs and ranked their combinations with all available partner drugs across selected O’Neil cell lines using MoCo-MultiSynergy prediction scores.

The top 10 predictions for paclitaxel and carboplatin are listed in Tables 5 and 6, respectively. Among the paclitaxel predictions, MK-8669 has been evaluated with paclitaxel in advanced solid tumors (Sessa *et al.* 2010), while MRK-003-mediated Notch inhibition enhanced the antitumor effect of paclitaxel in platinum-resistant ovarian tumor models (Groeneweg *et al.* 2014). For carboplatin, AZD1775 was repeatedly prioritized across several cell lines, consistent with studies of WEE1 inhibition combined with platinum- and taxane-based therapy (Leijen *et al.* 2016, Oza *et al.* 2020). Bortezomib has also been evaluated directly with carboplatin in recurrent ovarian cancer (Aghajanian *et al.* 2005). Overall, the case study shows that several highly ranked combinations are consistent with published evidence, whereas unsupported predictions remain hypotheses for experimental validation.

### 3.5 Feature representation visualization

To examine how the successive modules affect the learned sample representations, we applied t-SNE to features obtained at the four stages defined in Section 2.2 on the O’Neil dataset. For each drug–drug–cell-line sample, the representations of the two drugs and the corresponding cell line were combined before visualization. The four panels correspond to the representations obtained after multimodal feature initialization, momentum contrastive learning, heterogeneous hypergraph fusion, and synergy prediction, respectively.

**Figure 4:**
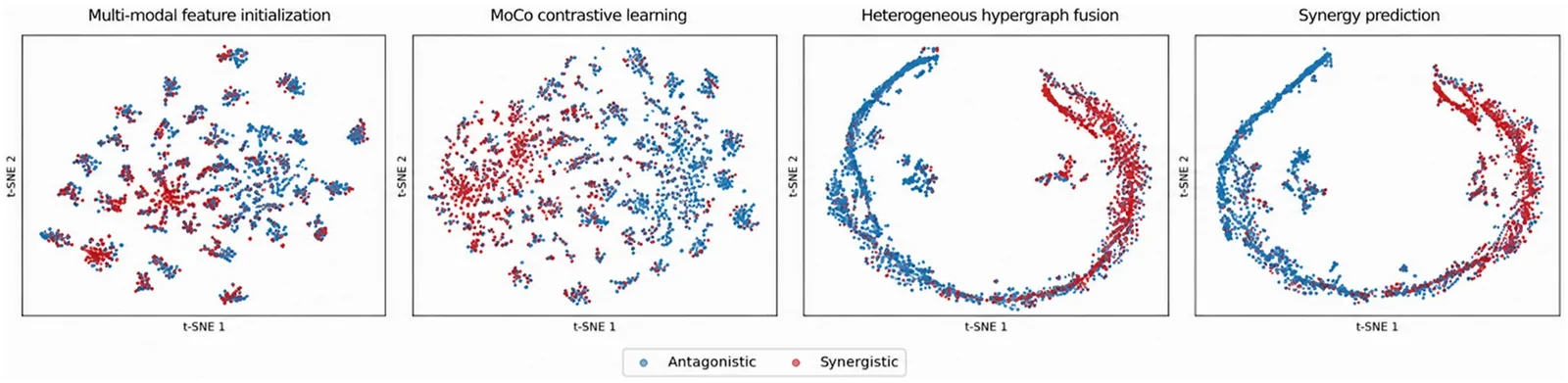
t-SNE visualization of sample representations obtained at successive stages of MoCo-MultiSynergy on the O’Neil dataset. From left to right, the panels show the representations after multimodal feature initialization, momentum contrastive learning, heterogeneous hypergraph fusion, and synergy prediction. The antagonistic samples and synergistic samples are represented by different clolors.

As shown in Fig. 4, the representations produced by multimodal feature initialization are dispersed and exhibit substantial overlap between antagonistic and synergistic samples. After momentum contrastive learning, the distribution becomes more structured, suggesting that consistency regularization reduces sensitivity to feature perturbations. Heterogeneous hypergraph fusion further improves the separation between the two classes by incorporating drug–drug–cell-line interactions and drug–disease associations. The representations supplied to synergy prediction show the clearest class separation, although some overlap remains. These visualizations provide qualitative support for the complementary roles of modality-specific contrastive learning and heterogeneous hypergraph refinement.

## 4 Conclusion

MoCo-MultiSynergy combines modality-specific momentum contrastive learning with heterogeneous hypergraph modeling for drug synergy prediction. The contrastive objectives regularize encoded drug and cell-line representations using augmented latent views, momentum-updated key projection heads and dynamic negative queues, while the heterogeneous hypergraph models synergistic drug–drug–cell-line triplets and drug–disease associations through gated residual propagation.

Across the O’Neil and NCI-ALMANAC datasets, MoCo-MultiSynergy matched or exceeded the compared methods under the Random mode and showed clearer performance advantages under the CLine and DrugComb modes. Component analyses demonstrated that the joint drug and cell-line MoCo objectives contributed substantially under the CLine and DrugComb modes, with additional benefits from hypergraph-based interaction modeling and gated residual refinement. The model also retained predictive ability in both leave-drug-out and cross-dataset validation, although the reduction in cross-dataset performance highlights the challenge posed by differences in scoring systems, experimental protocols and data distributions. The drug-centric analyses further showed that several highly ranked combinations were associated with published evidence for the same drug pairs or closely related treatment regimens.

Future work will incorporate drug–target, protein–protein and pathway relationships, extend the framework beyond drug pairs and improve generalization across screening platforms. MoCo-MultiSynergy provides a practical framework for prioritizing combinations for experimental evaluation.

## Supplementary Material

btag530_Supplementary_Data

## Data Availability

The datasets, preprocessing scripts, and source code underlying this article are available in the MoCo-MultiSynergy repository at https://github.com/27167199/MoCo-MultiSynergy.
