# Supplementary material for: Momentum contrast-enhanced multimodal representation learning for drug synergy prediction
Source: Bioinformatics. 2026 Jul 17;42(8):btag530. doi: 10.1093/bioinformatics/btag530 (PMC13433264; doi:10.1093/bioinformatics/btag530)
Supplement: Supplementary file 1 [file btag530_supplementary_data.docx]

**Momentum Contrast-Enhanced Multimodal Representation**

**Learning for Drug Synergy Prediction**

**(Supplementary Data)**

Yu Gu^1^ ,Xindi Huang^1^ ,Lifen Shi^1^, Leying Chen^1^,Guihua Duan^2^ ,and Cheng Yan^1^,∗

^1^School of Informatics, Hunan University of Chinese Medicine, 410208, Changsha, China.

* Contact. yancheng01@hnucm.edu.cn

# 1 Supplementary Methods And Implementation Details

## 1.1 Multimodal feature initialization

Drug molecules were converted from SMILES strings into molecular graphs, with atoms represented as nodes and chemical bonds as edges. A three-layer TransformerConv encoder with four attention heads generated atom-level representations, and global mean pooling produced a 256-dimensional graph-level drug embedding. Cell-line gene expression profiles containing 651 genes were projected into the same 256-dimensional latent space using a linear layer with tanh activation followed by two residual multilayer-perceptron blocks. Disease concepts were initialized from 768-dimensional CODER biomedical embeddings and projected into the common latent space. Disease representations were used as auxiliary contextual features and were not subjected to contrastive augmentation.

## 1.2 MoCo design and joint optimization

Momentum Contrast was used as an auxiliary self-supervised objective for the drug and cell-line modalities. For each encoded drug or cell line, feature masking and Gaussian perturbation generated two stochastic views of the same entity. The drug views were produced from encoded atom-level features before global pooling, whereas the cell-line views were generated from latent transcriptomic representations. One view was processed by a query projection head and the other by a momentum-updated key projection head. Representations of other entities stored in a first-in-first-out queue served as negative samples.

$$\theta_{k}\leftarrow m\theta_{k}+(1-m)\theta_{q}$$

The momentum coefficient was set to m = 0.999. Query and key projection heads used a 256-256-256 multilayer-perceptron architecture with ReLU activation and L2 normalization. A queue of K = 8192 normalized key representations was maintained independently for the drug and cell-line modalities. The InfoNCE objective increased agreement between the two views of the same entity while separating them from queued keys from other entities.

$$\mathcal{L}_{MoCo_{d}}=-\log\frac{exp(q^{d}\cdot k^{d+}/\tau)}{exp(q^{d}\cdot k^{d+}/\tau)+\sum_{n=1}^{K} exp(q^{d}\cdot k_{n}^{-}/\tau)}$$

The complete framework was optimized using binary cross-entropy for synergy prediction, an auxiliary relation-reconstruction loss, and the two modality-specific contrastive losses. The reconstruction branches used bilinear decoders to reconstruct predefined relationship matrices for drugs, cell lines and diseases from the refined node representations.

$$\mathcal{L}_{total}=\mathcal{L}_{BCE}+\alpha\mathcal{L}_{rec}+\beta(\mathcal{L}_{MoCo_{d}}+\mathcal{L}_{MoCo_{c}})$$

Training comprised 100 epochs of modality-encoder pretraining followed by joint optimization for up to 3000 epochs. The InfoNCE temperature was set to 0.07. During optimization, gradients updated the backbone and query branches, whereas the key projection heads were updated only by exponential moving average. The key branches and queues were used only during contrastive training.

Figure S1 summarizes the modality-specific MoCo procedure described above, which includes augmented-view generation, query and momentum-updated key projection, momentum-based key-parameter updates, negative-key queue maintenance and InfoNCE optimization for the drug and cell-line modalities. The two modality-specific modules operate on their respective representations and jointly contribute to the overall training objective.

## 1.3 Auxiliary relation reconstruction

An auxiliary reconstruction objective was used to preserve modality-specific pairwise relationships in the node representations after heterogeneous hypergraph refinement. Separate learnable bilinear decoders were used for the drug, cell-line and disease modalities. For modality r, the reconstructed relation matrix was calculated as:

$$\text{Â(}\text{r}\text{)=}\text{sigmoid}\text{[}\text{Z}\text{(}\text{r}\text{)}\text{W}\text{(}\text{r}\text{)}\text{Z}\text{(}\text{r}\text{)ᵀ]}$$

where Z(r) contains the refined node representations of modality r, W(r) is a learnable modality-specific bilinear transformation matrix and Â(r) is the reconstructed relation matrix.

For drugs, MACCS key fingerprints were generated from the SMILES representations using RDKit. The drug target matrix contained pairwise cosine similarities between the resulting molecular fingerprints. The continuous similarity values were retained directly as soft reconstruction targets, without thresholding, and diagonal entries were set to zero.

For cell lines, pairwise Pearson correlation tests were performed between the normalized 651-gene expression profiles. An entry in the cell-line target matrix was assigned a value of 1 when the two-sided Pearson correlation test produced a P-value no greater than 0.01 and was otherwise assigned a value of 0. For diseases, the same procedure was applied to the 768-dimensional CODER embeddings. Diagonal entries in both matrices were set to zero.

Binary cross-entropy was used to compare each reconstructed matrix with its corresponding target matrix. The complete auxiliary reconstruction loss was defined as the sum of the drug, cell-line and disease reconstruction losses:

$$\text{L}_{\text{rec}} = BCE[Â(d), A(d)] + BCE[Â(c), A(c)] + BCE[Â(s), A(s)]$$

The reconstruction loss was evaluated over the complete relation matrices. No negative-pair sampling, pair masking or additional similarity thresholding was applied. The three modality-specific losses contributed equally before the complete reconstruction loss was multiplied by the reconstruction weight α.

## 1.4 Hyperparameters

The main hyperparameters were selected by grid search using mean validation AUROC on the O'Neil dataset under the Random mode. Supplementary Table S1 lists the complete search spaces and the selected configuration. The contrastive weight β was evaluated over {0.001, 0.005, 0.02, 0.05, 0.1, 0.2}; β = 0.02 provided the best validation performance and was used in all main experiments.

**Supplementary Table S1.** Comprehensive hyperparameter search space and selected configuration for MoCo-MultiSynergy.

| **Hyperparameter** | **Search space** | **Selected value** |
| --- | --- | --- |
| Learning rate | {1e-3, 5e-4, 1e-4, 5e-5, 2e-5} | 1e-4 |
| Weight decay | {1e-1, 1e-2, 1e-3, 1e-4} | 1e-2 |
| Attention heads | {2, 4, 8} | 4 |
| Number of hypergraph layers | {2, 3, 4} | 3 |
| Reconstruction weight α | {0, 0.02, 0.05, 0.1, 0.2, 0.5, 1.0} | 0.05 |
| Contrastive weight β | {0.001, 0.005, 0.02, 0.05, 0.1, 0.2} | 0.02 |
| InfoNCE temperature τ | {0.01, 0.03, 0.05, 0.07, 0.10, 0.20, 0.50} | 0.07 |
| MoCo queue size K | {4096, 8192, 16384} | 8192 |
| Momentum coefficient m | {0.99, 0.996, 0.999} | 0.999 |

**Supplementary Figure S1.** Detailed workflow of the modality-specific Momentum Contrast modules. For each drug or cell line, two augmented views are processed by a query projection head and a momentum-updated key projection head. The two views of the same entity form a positive pair, whereas representations of other entities stored in modality-specific first-in-first-out queues serve as negative keys. The key projection heads are updated by exponential moving average, and the InfoNCE objectives regularize the drug and cell-line representations during joint model training.

# 2 Sensitivity Analyses

## 2.1 Sensitivity to the synergy-score threshold

The primary O'Neil experiments binarized Loewe synergy scores at 30. To assess sensitivity to this label definition, MoCo-MultiSynergy was retrained and evaluated under the Random mode using thresholds of 0, 10, 20, 30, 40 and 50. Supplementary Table S2 shows that AUROC remained high over a broad range of thresholds and reached its maximum at 30. AUPRC also peaked at 30 and declined at higher thresholds because fewer samples were labeled as synergistic, increasing class imbalance.

**Supplementary Table S2.** Sensitivity of MoCo-MultiSynergy to the Loewe synergy-score threshold on the O'Neil dataset under the Random mode.

| **Threshold** | **AUROC** | **AUPRC** |
| --- | --- | --- |
| 0 | 0.8259 | 0.6507 |
| 10 | 0.8617 | 0.6846 |
| 20 | 0.8975 | 0.7085 |
| 30 | 0.9433 | 0.7124 |
| 40 | 0.9389 | 0.5652 |
| 50 | 0.9345 | 0.4180 |

We additionally compared all methods across the same threshold range. Supplementary Figure S2 summarizes the method-wise trends, while Supplementary Tables S3 and S4 provide the corresponding AUROC and AUPRC values. Although the absolute metrics changed with the positive-label definition, MoCo-MultiSynergy remained competitive across all tested thresholds and achieved the highest AUPRC throughout the analysis.

**Supplementary Figure S2.** Performance of MoCo-MultiSynergy and the compared methods across different Loewe synergy-score thresholds on the O'Neil dataset. The left panel shows AUROC and the right panel shows AUPRC.

**Supplementary Table S3.** AUROC comparison under different Loewe synergy-score thresholds on the O'Neil dataset.

| **Method** | Th=0 | Th=10 | Th=20 | Th=30 | Th=40 | Th=50 |
| --- | --- | --- | --- | --- | --- | --- |
| DeepSynergy | 0.7955 | 0.8265 | 0.8865 | 0.9060 | 0.8990 | 0.8989 |
| NHP | 0.7611 | 0.8710 | 0.8700 | 0.8707 | 0.8600 | 0.8500 |
| DTF | 0.8037 | 0.8315 | 0.8728 | 0.9138 | 0.9087 | 0.9080 |
| HypergraphSynergy | 0.8063 | 0.8454 | 0.8773 | 0.9230 | 0.9194 | 0.9112 |
| HERMES | 0.8179 | 0.8486 | 0.8905 | 0.9330 | 0.9263 | 0.9264 |
| MARSY | 0.8041 | 0.9210 | 0.9220 | 0.9221 | 0.8659 | 0.8461 |
| JointSyn | 0.8130 | 0.8454 | 0.9268 | 0.9264 | 0.9216 | 0.9215 |
| MultiSyn | 0.8117 | 0.8506 | 0.8814 | 0.9281 | 0.9256 | 0.9171 |
| MoCo-MultiSynergy | 0.8259 | 0.8617 | 0.8975 | 0.9433 | 0.9389 | 0.9345 |

**Supplementary Table S4.** AUPRC comparison under different Loewe synergy-score thresholds on the O'Neil dataset.

| **Method** | Th=0 | Th=10 | Th=20 | Th=30 | Th=40 | Th=50 |
| --- | --- | --- | --- | --- | --- | --- |
| DeepSynergy | 0.4533 | 0.4679 | 0.4806 | 0.4920 | 0.3756 | 0.2985 |
| NHP | 0.5033 | 0.5550 | 0.5530 | 0.5540 | 0.4400 | 0.3000 |
| DTF | 0.5479 | 0.5671 | 0.5815 | 0.5920 | 0.4908 | 0.3711 |
| HypergraphSynergy | 0.5673 | 0.6066 | 0.6169 | 0.6280 | 0.5140 | 0.3623 |
| HERMES | 0.6091 | 0.6335 | 0.6623 | 0.6647 | 0.5241 | 0.4122 |
| MARSY | 0.5199 | 0.5800 | 0.5790 | 0.5786 | 0.4649 | 0.3280 |
| JointSyn | 0.5950 | 0.6188 | 0.6495 | 0.6479 | 0.5010 | 0.3996 |
| MultiSyn | 0.6063 | 0.6459 | 0.6624 | 0.6674 | 0.5328 | 0.3716 |
| MoCo-MultiSynergy | 0.6507 | 0.6846 | 0.7085 | 0.7124 | 0.5652 | 0.4180 |

## 2.2 Sensitivity to the InfoNCE temperature τ

The InfoNCE temperature was evaluated over {0.01, 0.03, 0.05, 0.07, 0.10, 0.20, 0.50} on the O'Neil dataset under the Random mode. Supplementary Table S5 shows that performance remained stable over a broad range of temperature values and declined slightly at larger values. The best AUROC and AUPRC were obtained when the temperature was set to 0.07. Therefore, a temperature of 0.07 was used in all experiments.

**Supplementary Table S5.** Sensitivity to the InfoNCE temperature **τ** on the O'Neil dataset under the Random mode.

| **τ** | **AUROC** | **AUPRC** |
| --- | --- | --- |
| 0.01 | 0.9423 | 0.7107 |
| 0.03 | 0.9421 | 0.7054 |
| 0.05 | 0.9417 | 0.7057 |
| 0.07 | 0.9433 | 0.7124 |
| 0.10 | 0.9423 | 0.7080 |
| 0.20 | 0.9410 | 0.7075 |
| 0.50 | 0.9405 | 0.7013 |

# 3 Additional Performance Evaluation And Statistical Analysis

## 3.1 Comparison with a random classifier

To verify that the learned models captured predictive signals beyond chance, all methods were evaluated against a random classifier under the Random mode. The random classifier assigned each sample an independently generated probability drawn from U(0,1). Supplementary Tables S6 and S7 report AUROC, AUPRC and F1 score on the O'Neil and NCI-ALMANAC datasets, respectively. MoCo-MultiSynergy substantially outperformed the random classifier and achieved the strongest overall results on both datasets.

**Supplementary Table S6.** AUROC, AUPRC and F1 score for all methods and the random classifier on the O'Neil dataset under the Random mode.

| **Method** | **AUROC** | **AUPRC** | F1 score |
| --- | --- | --- | --- |
| Random classifier | 0.5026 | 0.0922 | 0.1532 |
| DeepSynergy | 0.9060 | 0.4920 | 0.6824 |
| NHP | 0.8707 | 0.5540 | 0.6722 |
| DTF | 0.9138 | 0.5920 | 0.7148 |
| HypergraphSynergy | 0.9230 | 0.6280 | 0.7554 |
| HERMES | 0.9330 | 0.6647 | 0.8026 |
| MARSY | 0.9221 | 0.5786 | 0.6998 |
| JointSyn | 0.9264 | 0.6479 | 0.7742 |
| MultiSyn | 0.9281 | 0.6674 | 0.8134 |
| MoCo-MultiSynergy | 0.9433 | 0.7124 | 0.8501 |

**Supplementary Table S7.** AUROC, AUPRC and F1 score for all methods and the random classifier on the NCI-ALMANAC dataset under the Random mode.

| **Method** | **AUROC** | **AUPRC** | F1 score |
| --- | --- | --- | --- |
| Random classifier | 0.5018 | 0.1735 | 0.1386 |
| DeepSynergy | 0.8350 | 0.4912 | 0.6264 |
| NHP | 0.7769 | 0.3768 | 0.5127 |
| DTF | 0.8238 | 0.4679 | 0.6038 |
| HypergraphSynergy | 0.8530 | 0.5598 | 0.7246 |
| HERMES | 0.8537 | 0.5619 | 0.7448 |
| MARSY | 0.8357 | 0.5369 | 0.6925 |
| JointSyn | 0.8355 | 0.5197 | 0.7356 |
| MultiSyn | 0.8354 | 0.5148 | 0.7592 |
| MoCo-MultiSynergy | 0.8556 | 0.5675 | 0.7816 |

## 3.2 Statistical significance analysis

Two-sample t-tests were used to compare MoCo-MultiSynergy with HERMES using the AUROC and AUPRC values obtained from the five cross-validation folds. Supplementary Table S8 shows statistically significant improvements in both AUROC and AUPRC on the O'Neil dataset under all three modes. Supplementary Table S9 shows statistically significant improvements on NCI-ALMANAC under the CLine and DrugComb modes, whereas the differences under the Random mode were not statistically significant.

**Supplementary Table S8.** Statistical comparison between MoCo-MultiSynergy and HERMES on the O'Neil dataset.

| **Modes** | **HERMES** | | **MoCo-MultiSynergy** | | **p-value** | |
| --- | --- | --- | --- | --- | --- | --- |
|  | **AUROC** | **AUPRC** | **AUROC** | **AUPRC** | **AUROC** | **AUPRC** |
| Random | 0.9330 ± 0.0039 | 0.6647 ± 0.0088 | 0.9433 ± 0.0015 | 0.7124 ± 0.0038 | 2.44 × 10⁻³ | 6.00 × 10⁻⁵ |
| CLine | 0.8753 ± 0.0049 | 0.4350 ± 0.0102 | 0.9312 ± 0.0022 | 0.6708 ± 0.0045 | 9.54 × 10⁻⁷ | 2.16 × 10⁻⁸ |
| DrugComb | 0.8830 ± 0.0047 | 0.4900 ± 0.0100 | 0.9311 ± 0.0024 | 0.6813 ± 0.0049 | 9.83 × 10⁻⁷ | 3.20 × 10⁻⁸ |

**Supplementary Table S9.** Statistical comparison between MoCo-MultiSynergy and HERMES on the NCI-ALMANAC dataset.

| **Modes** | **HERMES** | | **MoCo-MultiSynergy** | | **p-value** | |
| --- | --- | --- | --- | --- | --- | --- |
|  | **AUROC** | **AUPRC** | **AUROC** | **AUPRC** | **AUROC** | **AUPRC** |
| Random | 0.8537 ± 0.0042 | 0.5619 ± 0.0098 | 0.8556 ± 0.0018 | 0.5675 ± 0.0042 | 3.92 × 10⁻¹ | 2.89 × 10⁻¹ |
| CLine | 0.7940 ± 0.0050 | 0.4650 ± 0.0108 | 0.8451 ± 0.0023 | 0.5454 ± 0.0050 | 1.58 × 10⁻⁶ | 8.91 × 10⁻⁶ |
| DrugComb | 0.7975 ± 0.0048 | 0.4300 ± 0.0105 | 0.8446 ± 0.0021 | 0.5403 ± 0.0055 | 2.42 × 10⁻⁶ | 7.47 × 10⁻⁷ |

# 4 Generalization Analyses

## 4.1 Leave-drug-out evaluation

Leave-drug-out evaluation was used to assess generalization to drugs absent from training. In the DrugSingle mode, each validation combination contained one unseen drug. In the more stringent DrugDouble mode, neither drug appeared during training. Supplementary Tables S10 and S11 show that MoCo-MultiSynergy outperformed HERMES and HypergraphSynergy in both modes on the O'Neil and NCI-ALMANAC datasets, respectively.

**Supplementary Table S10.** Leave-drug-out results on the O'Neil dataset.

| **Mode** | **Model** | **AUROC** | **AUPRC** |
| --- | --- | --- | --- |
| DrugSingle | MoCo-MultiSynergy | 0.8083 | 0.4572 |
| DrugSingle | HERMES | 0.7760 | 0.4042 |
| DrugSingle | HypergraphSynergy | 0.7260 | 0.3529 |
| DrugDouble | MoCo-MultiSynergy | 0.7648 | 0.4058 |
| DrugDouble | HERMES | 0.7316 | 0.3591 |
| DrugDouble | HypergraphSynergy | 0.6556 | 0.2983 |

**Supplementary Table S11.** Leave-drug-out results on the NCI-ALMANAC dataset.

| **Mode** | **Model** | **AUROC** | **AUPRC** |
| --- | --- | --- | --- |
| DrugSingle | MoCo-MultiSynergy | 0.7531 | 0.3105 |
| DrugSingle | HERMES | 0.7213 | 0.2721 |
| DrugSingle | HypergraphSynergy | 0.6784 | 0.2383 |
| DrugDouble | MoCo-MultiSynergy | 0.7135 | 0.2793 |
| DrugDouble | HERMES | 0.6839 | 0.2371 |
| DrugDouble | HypergraphSynergy | 0.6123 | 0.1974 |

## 4.2 Bidirectional cross-dataset validation

Cross-dataset validation was performed in both directions using drugs and cell lines shared by O'Neil and NCI-ALMANAC. As shown in Supplementary Table S12, training on O'Neil and testing on NCI-ALMANAC yielded an AUROC of 0.5922 and an accuracy of 0.7829, whereas training on NCI-ALMANAC and testing on O'Neil yielded an AUROC of 0.6879 and an accuracy of 0.8023. These results indicate that cross-dataset generalization was stronger in the NCI-ALMANAC-to-O'Neil direction.

**Supplementary Table S12.** Bidirectional cross-dataset validation results.

| **Training dataset** | **Test dataset** | **AUROC** | **Accuracy** |
| --- | --- | --- | --- |
| O'Neil | NCI-ALMANAC | 0.5922 | 0.7829 |
| NCI-ALMANAC | O'Neil | 0.6879 | 0.8023 |

# 5 Ablation Analyses

## 5.1 Component ablation

Supplementary Table S13 complements the AUROC ablation results reported in the main manuscript by providing AUPRC values under the Random, CLine and DrugComb modes. Removing both modality-specific MoCo objectives caused the largest reduction, particularly under the CLine and DrugComb modes. Removing either MoCo objective alone resulted in smaller changes, while excluding the heterogeneous hypergraph or gated residual mechanism also reduced performance.

**Supplementary Table S13.** Component ablation results (AUPRC) on the O'Neil dataset.

| **Strategy** | **Random** | **CLine** | **DrugComb** |
| --- | --- | --- | --- |
| MoCo-MultiSynergy | 0.7124 ± 0.0038 | 0.6708 ± 0.0045 | 0.6813 ± 0.0049 |
| w/o MoCo (drug + cell) | 0.6647 ± 0.0055 | 0.4035 ± 0.0080 | 0.4900 ± 0.0070 |
| w/o MoCo (drug) | 0.7102 ± 0.0039 | 0.6611 ± 0.0046 | 0.6742 ± 0.0050 |
| w/o MoCo (cell) | 0.7053 ± 0.0041 | 0.6608 ± 0.0047 | 0.6734 ± 0.0051 |
| w/o hypergraph | 0.6986 ± 0.0040 | 0.6543 ± 0.0047 | 0.6659 ± 0.0051 |
| w/o gated residual | 0.6955 ± 0.0042 | 0.6517 ± 0.0048 | 0.6625 ± 0.0052 |

## 5.2 Graph-level ablation of disease associations

To assess whether incomplete or noisy disease annotations introduced bias, all drug-disease edges were either removed or randomly permuted while preserving the total number of edges. Supplementary Table S14 shows only moderate performance changes relative to the complete model, indicating that the observed gains were not driven solely by the availability or assignment of disease edges.

**Supplementary Table S14.** Graph-level ablation results on the O'Neil dataset.

| **Configuration** | **Random** | | **CLine** | | **DrugComb** | |
| --- | --- | --- | --- | --- | --- | --- |
|  | **AUROC** | **AUPRC** | **AUROC** | **AUPRC** | **AUROC** | **AUPRC** |
| Full Model | 0.9433 ± 0.0015 | 0.7124 ± 0.0038 | 0.9312 ± 0.0022 | 0.6708 ± 0.0045 | 0.9311 ± 0.0024 | 0.6813 ± 0.0049 |
| No Disease | 0.9411 ± 0.0023 | 0.7068 ± 0.0046 | 0.9286 ± 0.0006 | 0.6368 ± 0.0032 | 0.9275 ± 0.0004 | 0.6382 ± 0.0028 |
| Shuffled Disease | 0.9394 ± 0.0007 | 0.7032 ± 0.0014 | 0.9271 ± 0.0011 | 0.6342 ± 0.0034 | 0.9234 ± 0.0017 | 0.6376 ± 0.0039 |

## 5.3 Drug-encoder architecture ablation

The TransformerConv-based drug encoder was replaced with GCN and GAT while all remaining components were kept fixed. Supplementary Table S15 shows that TransformerConv achieved the best AUROC and AUPRC under all three modes, supporting the use of attention-based molecular graph encoding for the drug modality.

**Supplementary Table S15.** Drug-encoder ablation results on the O'Neil dataset.

| **Drug Encoder** | **Random** | | **CLine** | | **DrugComb** | |
| --- | --- | --- | --- | --- | --- | --- |
|  | **AUROC** | **AUPRC** | **AUROC** | **AUPRC** | **AUROC** | **AUPRC** |
| TransformerConv (ours) | 0.9433 ± 0.0015 | 0.7124 ± 0.0038 | 0.9312 ± 0.0022 | 0.6708 ± 0.0045 | 0.9311 ± 0.0024 | 0.6813 ± 0.0049 |
| GCN | 0.9272 ± 0.0071 | 0.6853 ± 0.0102 | 0.9208 ± 0.0093 | 0.6534 ± 0.0124 | 0.9205 ± 0.0085 | 0.6618 ± 0.0110 |
| GAT | 0.9315 ± 0.0084 | 0.6934 ± 0.0056 | 0.9216 ± 0.0069 | 0.6583 ± 0.0058 | 0.9218 ± 0.0072 | 0.6675 ± 0.0061 |
